# Introduction of oral vitamin D supplementation and the rise of the allergy pandemic

**DOI:** 10.1186/1710-1492-5-8

**Published:** 2009-11-19

**Authors:** Matthias Wjst

**Affiliations:** 1 Institute of Genetic Medicine, EURAC research, Drususallee 1, I-39100 Bozen, Italy; 2 Helmholtz Zentrum München, Institute of Lung Biology and Disease, German Research Center for Environmental Health, Ingolstädter Landstr 1, D-85764 Neuherberg, Germany

## Abstract

The history of the allergy pandemic is well documented, enabling us to put the vitamin D hypothesis into its historical context. The purpose of this study is to compare the prevalence of rickets, vitamin D supply, and allergy prevalence at 50-year intervals by means of a retrospective analysis of the literature since 1880.

English cities in 1880 were characterized by an extremely high rickets prevalence, the beginning of commercial cod liver oil production, and the near absence of any allergic diseases. By 1930 hay fever prevalence had risen to about 3% in English-speaking countries where cod liver oil was preferentially used for the treatment of rickets. In 1980 vitamin D was used nation-wide in all industrialized countries as supplement to industrial baby food, thus eradicating nearly all cases of rickets. At the same time the allergy prevalence reached an all-time high, affecting about 30% of the population.

Time trends are therefore compatible with the vitamin D hypothesis although direct conclusions cannot be drawn. It is interesting, however, to note that there are at least two earlier research papers linking synthesized vitamin D intake and allergy (Reed 1930 and Selye 1962) published prior to the modern vitamin D hypothesis first proposed in 1999.

## 

The vitamin D allergy hypothesis [1] attributes the initial sensitization against allergens during the newborn period to immunological side effects of vitamin D supplements used for rickets prevention. The increasing interest in the vitamin D hypothesis is understandable because all other hypotheses about the origin of the allergy epidemic have largely failed to provide any clear answers. Moreover, none of the current hypotheses have ever been tested for compatibility with the historical development of the allergy pandemic.

It may therefore be interesting to examine historical data on vitamin D intake and prevalence of allergy. As chosen method, a systematic analysis of articles published in *Pubmed *since 1950 was combined with a full-text search of all issues of *Science *and *Nature *since 1869. Furthermore, current Google book content was searched in addition to a manual search of textbooks for the keywords vitamin D (and chemical analogues) and allergy between 1920 until 1950 (see also acknowledgments).

*1880*. Allergic manifestations were so rare in 1880 that today they would be considered an "orphan disease". This may reflect a recognition bias in a community that was understandably preoccupied with more pressing, life-threatening conditions such as cholera, tuberculosis, typhoid and measles. Nevertheless, allergic symptoms were clearly described at that time. The few studies on allergic diseases from the 19th century all rely on a limited number of cases. The British doctor Harrison Blackley wrote in his 1873 book "Hay Fever: Its causes, treatment, and effective prevention": "Even in this country, where the disorder probably had its commencement and where it is still more common than in any other part of Europe, there are medical men to be found who know very little about it; and on the Continent there are still some to be found who have never even heard of the disease" [2]. The origins of the disease are vague [3]. The first formal description of hay fever is usually ascribed to John Bostock, who presented his own case in 1819 to the London Medico-Chirurgical Society [4]. Another description was made in 1859 when the German professor Philipp Phoebus from Giessen published the first large allergy study [5], which was based on 158 cases. The sample consisted of patients from many hospitals because allergy was such a rare disease. In 1876 the American physician George Beard, a contemporary of Blackley, assembled only 100 patients [6]. At the end of the 19th century, allergy prevalence may therefore be estimated at 0.1% in England, as well as in the United States of America [3]. In continental Europe, it was not until 1906 that the term "Allergie" was introduced by Clemens von Pirquet.

In 1880, vitamin D was not yet known. It was not until 30 years later that the antirachitic property of cod liver was firmly established by Edward Mellanby, an English physician, and Elmer McCollum, an American biochemist [7,8]. However, already in the mid 19th century there existed a "folk tradition in some areas that cod liver oil was a specific, potent preventive, and this was endorsed by many physicians" [9]. This tradition was restricted to the coasts of England, Holland, and France [10]. Industrialized cod liver oil production (as the sole source of vitamin D food supply at that time) did not start before 1853 in Norway, Greenland, and Iceland according to a historical review [11]. The reason may be the way cod liver oil was produced. Because cod livers and gall-bladders could not be sold on the fish market, the fishermen placed this garbage in barrels in front of their houses until the end of the season. When the rotten livers were in an advanced stage of putrefaction, a deep yellow, creamy oil was skimmed off. Color, smell and taste, however, prevented any major distribution until 1853, when a steam-based extraction was invented. The new procedure produced a more or less colorless oil marketed as the "Finest steam-prepared Lofotan Cod-Liver Oil" [11]. Most of the oil was sent either to Hammerfest or to Bergen, from where it was then exported to Hamburg, London and New York and marketed as an aid for physical fortification and constitutional improvement. An advertisement for a cod liver oil medicine in the April 18, 1890 issue of Science proclaimed the "prevention or cure of coughs and colds in both the old and young" - effects rediscovered only in 1941 [12] and again in 2006 [13].

Rickets was a common disease in Europe and North America at the end of the 19th century. For this reason, British Medical Association [14] conducted a large survey on the disease and concluded that "first, its great frequency in large towns and thickly peopled districts, especially where industrial pursuits are carried on, and its comparative rarity in rural districts; secondly, the greater tendency to rickets in the rural parts of the south of Great Britain than in those of the north (...) In Norway and Denmark it has a subordinate place in the statistics of sickness relating to the earliest year of life. Its principal seats are Germany, England, Holland, Belgium, France and Northern Italy while southern Italy, the southern provinces of Spain, and still more Turkey and Greece, enjoy a notable immunity from it." Most cases were certainly mild, however, with many people being affected in endemic areas. According to several contemporary books, symptoms in new-born children usually appeared as early as the third or fourth month with head sweating and craniotabes. Rickets showed a seasonal incidence peak with most cases being born in fall. Theories of the origin were manifold [10] ranging from genetics, over-nutrition, poor diet, acidosis, a manifestation of syphilis or other infectious origin, a thyroid, parathyroid or adrenal gland disease, some noxious gases, "lack of hygiene", and prevention of sunlight. Even the animals in the London Zoological Gardens were affected by rickets; pictures in the National Gallery in London today still show rachitic children [15]. In some English districts more than half of the population had been affected by rickets - it was so highly prevalent that it became known as "English disease" in continental Europe. The situation in English cities in 1880 may therefore be characterized by a high rickets prevalence, the early beginning of industrial production of cod liver oil, and rather absent allergic disease.

*1930*. It is frequently assumed that there was low allergy prevalence in 1930 although this view is not supported by contemporary reports. Due to increasing patient demands, in 1897 the German *Heufieberbund *(Hay Fever Association) was founded on Helgoland. By 1928 the prevalence of hay fever had risen from a few affected people to approximately 1% [16], which is consistent with data from an (unpublished) letter of the former president of the Hay Fever Association to the director of the Robert Koch Institute in Berlin in 1935. For the U.S., Scheppegrell reported [3] about 1.2 million hay fever sufferers in 1922. Only a short time period later that number increased to between 4 and 5 million, corresponding to a prevalence of 3% [17]. Besides this first increase in allergy prevalence, the most remarkable observation was a shift from the more aristocratic part of the society being affected to a disease now occurring in all social classes: "each one of us has the plague within him; no one on earth, is free from it" (from Camus' novel "The plague"). A high social class may have meant better medical treatment and availability of vitamins while the extension to lower social classes may have indicated that ordinary people could now also afford vitamin supplements.

As Jackson notes in his book "Allergy - The History of a Modern Malady" [18], allergy prevalence changed only slowly during the following years before reaching today's epidemic proportions in the Western world. "In Switzerland, for example, the prevalence of hay fever rose from an estimated 0.82 percent in 1926 to 5 percent in 1958, and to approximately 10 percent by the 1980s. Beyond Europe, epidemiological studies provided ample evidence of rising trends in most allergic diseases during the second half of the twentieth century, especially in New Zealand, North America and Australia." [18]

This apparent increase in allergy prevalence is paralleled by a steady increase in cod liver oil production. In 1927, Norway exported most of its cod liver oil to the United States (35,127 hectoliters), Great Britain (16,000 hectoliters) and Germany (9,537 hectoliters). Furthermore, vitamin D metabolites could be chemically characterized due to the groundbreaking work of Adolf Windaus in the early 1920s [19], with the antirachitic effect of vitamin D firmly established by Mellanby and McCollum [8]. Because rickets was so widespread in the English-speaking countries, they had a great interest in supplementing their population with cod liver oil. In addition, 1927 Vigantol, an irradiated ergosterin, was introduced into the market by E. Merck AG, who produced between 2 and 3 kg of vitamin D2 per year. Production increased after 1938 up to 40 kg but ceased in 1945 [19] and was started again in 1949.

Merck AG also sold a combination of vitamin A and vitamin D (that was already on the market in the pre-war years). This combination, mimicking the high vitamin A and vitamin D content of cod liver oil, seems to antagonize the immunological effects of vitamin D [20,21]. The effects of cod liver oil may therefore not be fully comparable to chemically synthesized vitamin D. Another difference is the oily basis of cod liver extract that may have less allergenic properties than the watery solution used for some vitamin D supplements [22]. Finally, cod liver oil was given to toddlers only and not to newborn children whose immune system is adapting to the environment [23].

In England in 1930, however, the prevalence of rickets was still high, with air pollution prohibiting the natural supply of vitamin D through exposure to sunlight. During the 1930s and '40s "smog" was the term coined for the London type of air pollution. Furthermore, prevalence statistics for rickets in Germany in 1930 may be obtained from a survey in Munich showing that 38% of the children had at least minor signs of rickets [24]. With UV lamps, cod liver oil and synthetic vitamin D, however, a cure for rickets had been found. It was not until later that vitamin D was also increasingly used for the prevention of rickets. In summary, hay fever prevalence in 1930 was still around 3% in the industrialized countries, but a first major increase was already apparent in English-speaking countries. Cod liver oil was used for the treatment of rickets, but it was not used prophylactically in the newborn period until the following decades.

*1980*. The prevalence of allergy seemed to level off during the postwar years with another increase in the late 1970s [25]. During the late 1980s standardized prevalence data in children (ISAAC) [26] and adults (ECRHS) [27] have been obtained showing clearly that the highest prevalence was in industrialized countries. This is also the result of a more recent analysis that showed a year-round high level of allergic diseases in English-speaking countries [28].

Vitamin D prophylaxis had been more or less discontinued during the war years in Germany [29], where the vitamin D supply was difficult. It was not until 1950 that rickets prophylaxis was introduced by midwives in Bavaria and then slowly adopted by other German states [29]. When several cases of hypervitaminosis occurred due to high amounts of vitamin D given at that time, the president of the German Society of Pediatrics issued a warning on using a standard prophylactic scheme. Despite this warning, in 1971 a central childhood examination program was established which included daily oral doses of vitamin D3 between 500 IU and 1000 ID per day.

Vitamin D is now even sold over the counter. Moreover, it is included in most commercial baby food products although the substance itself has never undergone the rigorous preclinical testing one would expect for a chemically synthesized prohormone. Dosing is still largely done as an "equivalent of a tablespoon of cod liver oil".

Nevertheless, due to the effective use of vitamin D, rickets nearly disappeared in England [30]. Although there were some minor outbreaks of rickets in the 1960s, these were mostly observed in dark-skinned immigrants. For more than a decade the total number did not exceed 675 cases from all hospitals in England and Wales [15]. The number of children hospitalized for rickets has dropped to negligible numbers compared to the huge public health problem that rickets presented only a few decades ago. The same is also true for Germany, where rickets no longer plays a major role. Between 1978 and 1988 only 100 cases were observed in the city of Berlin [29].

In summary, between 1980 and 2000 allergy prevalence reached an all-time high with up to 30% of the population being affected. Today, although cod liver oil is still in use, most newborn food now includes vitamin D2 or D3, leading more or less to the extinction of clinically manifest rickets.

### Connecting the dots

A temporal coincidence of an exposure and outcome like the reported association above cannot provide any proof of causality although it may be seen as another piece in the puzzle.

On the other hand the absence of a temporal coincidence would provide a strong argument against any given hypothesis. Unfortunately, the prevailing hygiene hypothesis has never been tested to explain the historical development, although there would have been many characteristic features that could have been tested like the number of siblings, day-care use, or farm exposure. It has been repeatedly claimed that drinking unpasteurized milk will protect against allergy [31], while the history of pasteurization makes such a relationship unlikely [32]. Pasteur developed it in 1864, the procedure was introduced in 1889, and already by 1920 all commercial milk underwent pasteurization in the U.S. Pasteurization therefore cannot be the culprit, although avoidance of pasteurized (and additionally vitamin supplemented milk) may indeed relate to lower allergy rates. It is also unlikely from a historical standpoint that low vitamin levels in the population may be related to allergy as claimed by one group [33,34].

Another question is whether an association of vitamin D and allergy may have been noted earlier - for example when neither vitamin D nor allergies were so widespread. Indeed, there are at least two earlier reports. In 1932 Reed [35] cited a reference to a researcher who " ... employed rachitic rabbits and compared their reactions to those hypervitaminized with vigantol. It was found that in both groups there was impairment of formation of complement-fixing antibody but that the formation of precipitin and hemolysin was enhanced. Both conditions intensified active and passive anaphylactic reactions. In general, the changes were slightly more marked in hypervitaminosis than in deficiency." Another report is by the eminent Hans Selye 1962, who worked with rats pre-treated with a vitamin D analogue before they were sensitized with egg white. He described the resulting reaction as "calciphylaxis" (in analogy to "anaphylaxis" - immunoglobulin E was not discovered until 6 years later). A related editorial in Science [36] made clear that "the investigators who repeat Selye's laboratory work will raise this question about the major premise of calciphylaxis: Is it an allergic or hypersensitive state (a manifestation of altered responsiveness dependent upon a sensitiser and a challenging agent) or is it better defined by some other concept?"

## Conclusion

It was not until 1999, when the basic process of sensitization was associated with the immune effects of oral vitamin D supplements [1], that researchers arrived at a preliminary answer to this question. A series of clinical and epidemiological studies [37-42] provided further evidence, although this may still not be the final answer. More recent unpublished work shows that there may be a complicated relationship between external supply, endogenous production, metabolism, signalling pathways, and the development of allergy. Having identified, however, a factor repeatedly associated with allergic sensitization, this gives us the opportunity to think about rational strategies for allergy prevention including randomized clinical trials are now planned or already underway.

## Competing interests

The author declares that they have no competing interests.

## Authors' contributions

The author did the literature search and wrote the paper.
